# Fostering Collective Intelligence in Human–AI Collaboration: Laying the Groundwork for COHUMAIN

**DOI:** 10.1111/tops.12679

**Published:** 2023-06-29

**Authors:** Pranav Gupta, Thuy Ngoc Nguyen, Cleotilde Gonzalez, Anita Williams Woolley

**Affiliations:** ^1^ Gies College of Business University of Illinois, Urbana‐Champaign; ^2^ Department of Social & Decision Sciences Carnegie Mellon University; ^3^ Tepper School of Business Carnegie Mellon University

**Keywords:** Human–AI collaboration, Collective intelligence, Sociocognitive architectures, Cognitive architectures, Artificial social intelligence, Instance‐based learning

## Abstract

Artificial Intelligence (AI) powered machines are increasingly mediating our work and many of our managerial, economic, and cultural interactions. While technology enhances individual capability in many ways, how do we know that the sociotechnical system as a whole, consisting of a complex web of hundreds of human–machine interactions, is exhibiting collective intelligence? Research on human–machine interactions has been conducted within different disciplinary silos, resulting in social science models that underestimate technology and vice versa. Bringing together these different perspectives and methods at this juncture is critical. To truly advance our understanding of this important and quickly evolving area, we need vehicles to help research connect across disciplinary boundaries.

This paper advocates for establishing an interdisciplinary research domain—Collective Human‐Machine Intelligence (COHUMAIN). It outlines a research agenda for a holistic approach to designing and developing the dynamics of sociotechnical systems. In illustrating the kind of approach, we envision in this domain, we describe recent work on a sociocognitive architecture, the transactive systems model of collective intelligence, that articulates the critical processes underlying the emergence and maintenance of collective intelligence and extend it to human–AI systems. We connect this with synergistic work on a compatible cognitive architecture, instance‐based learning theory and apply it to the design of AI agents that collaborate with humans. We present this work as a call to researchers working on related questions to not only engage with our proposal but also develop their own sociocognitive architectures and unlock the real potential of human–machine intelligence.

## Abstract 2

How do we know that a sociotechnical system, as a whole, is exhibiting collective intelligence? We outline a research agenda for COHUMAIN by proposing sociocognitive architectures as a vehicle for designing and developing the dynamics of sociotechnical systems and illustrate this holistic approach by discussing the transactive systems model of collective intelligence and instance‐based learning.

## 1 Introduction

Work on artificial intelligence (AI) has grown at an exponential pace. From feedback‐loop cybernetics and generalized cognitive architectures to reinforcement learning models that organize knowledge and iteratively improve themselves, we now have large‐language models capable of leveraging the deep patterns in our collective knowledge to generate new insights. As the scope and penetration of AI‐powered machines explode, the volume of information and pace of change is outstripping our bounded cognitive capacity. Hence, we have come to rely on physical and digital machines to mediate our collective actions—augment our memory, manage our attention, and help us coordinate our collective decisions. In adjacent fields, social scientists are refining their understanding of collective intelligence (CI) in human systems, broadly defined as a group's ability to solve various problems across different environments (Riedl, Kim, Gupta, Malone, & Woolley, 2021; Woolley, Chabris, Pentland, Hashmi, & Malone, 2010). We have moved from simply harnessing the wisdom of crowds for solving prediction problems to exploring how diversity and group structure help humans distributed across the globe to organize dynamically and solve collaboration problems.

While significant progress has been made in the development of AI, as well as the social scientific development of CI, what has developed more slowly is the holistic, integrated understanding of human–machine systems. *How do we know that such a sociotechnical system as a whole, consisting of a complex web of hundreds of human–machine interactions, is exhibiting CI?* Extant research on human–machine interactions occurs in different disciplinary silos and focuses primarily on the phenomena of interest to the specific discipline. Consideration of adjacent domains is secondary and results in the implementation of technical systems that produce “unexpected” adverse outcomes that were arguably foreseeable had they been developed in a more interdisciplinary environment. We assert that creating vehicles to integrate the sciences at this juncture *is critical*. This needs to occur during the earlier phases of development rather than at later stages when we are left to deal with the unintended consequences. Not doing so opens us up to not just organizational or market inefficiencies but also significant societal risks. Given the cumulative nature of this work, understanding how to design and implement effective human–machine collaboration can have important implications for the design of future intelligent systems and artificial general intelligence. Unfortunately, we lack a vehicle for systematically translating and integrating insights across fields into a shared and holistic frame (see related efforts in Galesic et al., 2023).

In this paper, we address this gap by proposing a research agenda for *Collective Human‐Machine Intelligence* (COHUMAIN)—an interdisciplinary research domain to facilitate the development of holistic models that inform the design and study of collaboration dynamics in sociotechnical systems. The initial development of the field of AI benefited significantly from the variety of cognitive architectures that articulated the key components and processes of an individual agent's decision‐making (e.g., general problem solver by Newell, & Simon, 1972; ACT‐R by Anderson, Conrad, & Corbett, 1989). Drawing a parallel to cognitive architectures, our main claim is to use sociocognitive architectures to study this issue systematically, integrate interdisciplinary knowledge, and push COHUMAIN research for multiagent systems. We identify four problems unique to this endeavor and recommend two features necessary for sociocognitive architectures. And briefly review extant work on human–machine interaction, human–AI trust, and machine theory of mind that address our understanding of these issues so far.

We then present a new sociocognitive architecture, the transactive systems model of collective intelligence (TSM‐CI; Gupta & Woolley, 2021), which articulates the three functional systems governing collective memory, attention, and reasoning that form the core of any intelligent sociotechnical system, including the domain of collective human–machine intelligence. We extend the transactive systems model to COHUMAIN by discussing how AI agents can augment collective memory, attention, and reasoning systems. Finally, as a third contribution, we highlight the value of designing AI agents with cognitive architectures that align with the encompassing sociocognitive architecture. Specifically, discussing instance‐based learning theory (IBLT; Gonzalez, 2013; Gonzalez, Lerch, & Lebiere, 2003), a cognitive architecture for developing AI agents compatible with the TSM‐CI.

By proposing COHUMAIN and illustrating a sociocognitive architecture as a vehicle for integrating disciplinary perspectives, we wish to spark the interest of researchers across fields to not only engage with our proposal but also develop their own sociocognitive architectures and unlock the real potential of human–machine intelligence.

## 2 COHUMAIN: A holistic and interdisciplinary approach to design of sociotechnical systems

Tapping into the true potential of human–AI collaboration requires a systems‐level comprehension of how humans and machines coordinate interdependent actions in response to their environment and how humans and machines make sense of each others’ cognitive states and resources that guide their said interdependent actions. This understanding will require the integration of social science and AI as well as the integration of traditional research and applied technical design, as the scientific and applied approaches can iteratively build knowledge together more quickly to advance progress. The scientific analysis of the system's emergent behaviors guides architectural design choices, which in turn changes system behavior.

Advocacy for a systems‐level approach dates back to Newell (1973), who argued for the value of systems‐level research as a vehicle for integration in cognitive science and AI, claiming “You can't play 20 questions with nature and win.” A cognitive architecture provides a theoretical framework to unify many relationships that enables the testing of multicausal theories rather than the more narrowly scoped questions of traditional research that test one or two causal links. Cognitive architectures enable researchers to refine systems theory by testing claims, playing out implications, and iteratively shaping the architectural design for additional investigation cycles.

Building on the intellectual heritage of cognitive architectures, we propose that researchers collaborate to develop systems‐level sociocognitive architectures to advance research in COHUMAIN. Doing so will help advance COHUMAIN by providing common ground for integrating disciplinary perspectives. Just as a cognitive architecture specifies the underlying infrastructure, components, and functional processes of an individually intelligent agent (Anderson & Lebiere, 2014; Langley., Laird, & Rogers, 2009), a sociocognitive architecture specifies the underlying infrastructure, components, and functional processes for a multiagent, sociotechnical system, particularly a complex adaptive system that is capable of general problem‐solving. That is, exhibit collective intelligence, as CI is broadly defined as the ability of any group to solve a broad range of problems or maintain performance in a continuously changing environment (Gupta, 2022; Riedl et al., 2021; Woolley et al., 2010).

Unfortunately, there is no straightforward method for building and combining individual‐level cognitive architectures into the collective, sociocognitive architectures needed to support an integrated interdisciplinary approach to COHUMAIN research. Cognitive architectures aim to build autonomous general problem solvers or AI by asking how an autonomous agent perceives, understands, and acts in the environment productively. By contrast, sociocognitive architectures ask how *multiple* autonomous agents (humans and AI agents) collaborate and problem‐solve *together*. This involves working interdependently by collectively perceiving, thinking, and acting together in the environment productively. Progress on the former does not guarantee progress on the latter, as sociocognitive architectures require both an understanding of how individual agents process information and make decisions as well as how, in a collective context, they affect one another and adapt to complement the processes of other agents and serve to maintain the coherence of the system as a whole. Thus, cognitive architectures provide an important and complementary input. Yet, we need to build on them using a slightly different approach for COHUMAIN research, one that captures the nature of the complex adaptive systems required for CI.

**Fig. 1:**
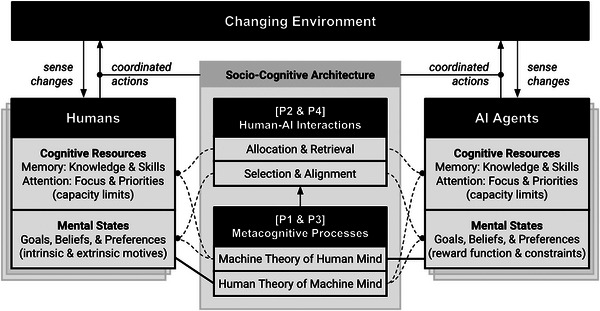
Schematic for the design of the sociocognitive architecture of sociotechnical systems. The two generic features of a minimal sociocognitive architecture are highlighted: metacognitive processes (P1 and P3) and human–AI interactions (P2 and P4). Note. It only depicts the schema for human–AI interactions and does not show human–human and AI–AI schemas.

We assert that any sociocognitive architecture, even one that is minimally scoped, will need to address four core problems to enable the alignment and coordination between humans and AI agents necessary for CI's emergence. The four core problems (P1–P4 depicted in Table 1 and Fig. 1) reveal two categories of processes that are necessary for any sociocognitive architecture that exhibits CI. First, collaborators engage in metacognitive processes to access each others’ mental states and collective cognitive resources, that is, develop a reasonable theory of mind (ToM) (P1 and P3). Second, through interacting with one another, collaborators gain information about environmental changes and dynamically evolve shared norms and routines to align mental states and coordinate collective cognitive resources (P2 and P4). The successful result of these processes will be the formation of collective cognition, whereby collaborators vastly expand their collective cognitive capabilities. The effective enactment of these two sets of processes will serve to build a foundation of trust in the human–AI system, as perceiving the expansion of collective capability will contribute to the cognitive bases of trust and observing ongoing engagement in joint activity to achieve shared goals will contribute to the affective bases of trust (Glikson & Woolley, 2020).

**Table 1:** Four core problems (P1–P4) underlying the emergence of collective intelligence in human–machine systems: Formulating a research agenda for the design of sociocognitive architectures for COHUMAIN (collective human‐machine intelligence)

	Between‐Member Metacognitive Processes	Between‐Member Interactions
Mental states	**P1**. How do individual members perceive and represent each others’ mental states (e.g., goals, beliefs, preferences)? How does doing so shape their own mental states and support the emergence of collective cognition?	**P2**. Given diverse and changing mental states, how do members engage in trustworthy interactions to dynamically align their mental states and select joint priorities that maximize collective outcomes?
Cognitive resources	**P3**. How do individual members perceive and represent each others' cognitive resources (e.g., specialized knowledge and skills, information‐processing capacity)? How does recognizing self‐other differences in cognitive resources facilitate the development of collective cognition?	**P4**. Given distributed and changing cognitive resources, how do members develop and engage shared norms of interactions to dynamically coordinate interdependent actions that ensure efficient utilization of collective resources?

A recent information‐theoretic approach that models CI from first principles finds that agent features consistent with ToM and goal alignment are necessary conditions for multiple agents to optimize their joint outcomes (Kaufmann, Gupta, & Taylor, 2021). Thus, *we claim that any sociocognitive architecture developed to address COHUMAIN needs to specify mechanisms for (1) between‐member metacognition and (2) the development of engagement rules that enable coordinated action and trust development*.

It is important to note that a key assumption we make here is that all autonomous machines or AI agents are “designed systems” and hence, unlike humans, do not possess higher order goal autonomy.^1^ While one of their key goals is to serve humans by playing different roles, they are likely to have other goals (e.g., profit) that serve the designers of the AI that are not part of the collective. As such, we do not claim AI agents to be humans’ cultural peers. They may be considered part of the technological context with features that allow humans to interact with them as distinct entities or “anthropomorphized” team members (e.g., Siri or ChatGPT). This assumption about AI agents’ goal autonomy may or may not hold in the future. Nevertheless, we think this is a reasonable assumption. In most organizational cases, even humans (i.e., employees) do not simply act on their intrinsic motivations. Their goals must be aligned with or subordinated by their organizations. And the extent of this alignment affects their ability to perform. In this way, the core problem of coordinating the members' cognitive states (goals, preferences, and beliefs) still needs to be resolved to achieve CI. Humans and AI agents together form the fabric of the sociotechnical system.

In practice, the AI agents' goals are likely to become more complex over time. While their development is guided by the goals of their designers, at the same time, if they are intended to learn and adapt to enhance the capability of a human–AI system, those goals will also be influenced through interaction with human collaborators. The degree to which an AI agent imposes its goals versus adapting to human collaborators’ goals will depend on the role they were designed to play. Gupta and Woolley (2021) discuss three types of roles AI agents can play in a collective: (1) assistive AI that scaffolds or augments individual cognition, (2) coach AI that facilitates and nudges collective cognition, and (3) diagnostic AI that monitors emergent collective behavior. Clearly, for each of these roles, an AI agent is initially endowed with a set of desired goal states which guide how they assist, nudge, or manage human users; however, across these roles, AI assistants will likely adapt to the goals and needs of their human user while AI coaches could be more assertive in finding ways to shape human behavior by adjusting how they encourage the human(s) to move toward desired goal states. As the capability of AI agents grows, they will become more skillful in influencing human action in the direction of their own goal states. Unless there are clear mechanisms for humans to influence their goals, we will stand to lose as a society and exhibit lower CI due to the one‐directional shaping of collective cognition.

In summary, the development of sociocognitive architectures for COHUMAIN will depend on researchers’ understanding of how to best design agents to facilitate effective collaboration. We discuss two categories of processes that are necessary for any sociocognitive architecture. Next, we review insights for a few relevant areas of research and then describe our take on a candidate sociocognitive architecture, the TSM‐CI, and a compatible learning‐based cognitive architecture.

## 3 Review of extant research on foundations for COHUMAIN

A few bodies of research are particularly relevant to COHUMAIN and provide preliminary principles for developing sociocognitive architectures. These works of literature include human–machine interaction, human–AI trust, and machine ToM.

### 3.1 Human and machine interaction

Research on the ways that humans and technology interact has been ongoing for several decades, including work in areas such as human‐computer interaction (HCI) and human–autonomy integration (HAI; O'Neill, McNeese, Barron, & Schelble, 2020; Schelble, Flathmann, & McNeese, 2020). HCI has traditionally focused on how humans use and interact with computing devices, with an initial focus on interface design, and has evolved relatively sophisticated models in some areas to deal with multimodal inputs and outputs, and ways for algorithms to read and adapt based on human cues (G. J. Kim, 2015). HAI, by contrast, has been less focused on systems or interface design and more on how humans and automated systems interact and collaborate, by some accounts beginning as far back as the 1980s (O'Neill et al., 2020). In much of the HAI work, however, technology has been viewed as an aid that is subservient to humans, with frameworks for considering a technology's level of ability serving as a means for assessing the ways collaboration could evolve as technological capability increases (Endsley, 2017).

Recent research on human–autonomy teaming (HAT) focuses on teams in which humans and autonomous AI agents function as coordinated units to achieve a common goal (McNeese, Demir, Cooke, & Myers, 2018) and more directly addresses the ways that technology can collaborate with humans as a teammate (O'Neill et al., 2020; O'Neill, Flathmann, McNeese, & Salas, 2023) compared to other extant research on human–AI interaction. Across much of HAT research, human–AI team goals are associated with a reward that is equally shared among the agents (Matignon, Laurent, & Le Fort‐Piat, 2012), but the autonomous agents have been primarily designed to carry out independent performance on a specific task. Thus HATs are often trained to function as the sum of individual parts rather than as highly interdependent collaborators (Burke, Murphy, Coovert, & Riddle, 2004; Salas, Bowers, & Cannon‐Bowers, 1995, 2008; Tsifetakis & Kontogiannis, 2019). That is, computational modeling of autonomous agents has been mostly used to model how teams operate via stable, pre‐programmed processes instead of as adaptive, complex, and self‐organizing systems (O'Neill et al., 2020). Recognizing the potential value of greater integration of human and AI teammate inputs, there are increasing calls for the development of models to enable human and autonomous agents to interact in team‐like structures to achieve common objectives (Cooke, Demir, & McNeese, 2016; Glikson & Woolley, 2020; Larson & DeChurch, 2020; M. D. McNeese & McNeese, 2020; Myers et al., 2019). Consequently, some argue that to be considered a legitimate team member, the autonomous agents must have both process and outcome interdependence with the human team members (Lyons, Mahoney, Wynne, & Roebke, 2018; Wynne & Lyons, 2018).

In related research, some HAT studies examine the question of the level of autonomy agents should have, and the results are mixed. For instance, some studies report that humans perceive autonomous teammates to be easier to work with and their collaboration to be better when the agents have high levels of autonomy (Azhar & Sklar, 2017; Johnson et al., 2012; J. L. Wright, Chen, & Barnes, 2018; J. L. Wright, Chen, Quinn, & Barnes, 2013). Others report that a moderate level of agent autonomy was perceived as more effective compared to a lower or higher level (M. C. Wright & Kaber, 2005). Some studies suggest that an important moderator of the effect of the level of agent autonomy is the human participants’ level of ability. For instance, participants with low spatial ability experienced the greatest performance benefits from increases in agent autonomy (Chen et al., 2013; J. L. Wright et al., 2013, 2018). Therefore, a key benefit of HATs is achieved when they can assess human teammates and adjust to variations in their abilities. The ability to assess the human teammate has also been identified as one important aspect of agents’ situation awareness in the context of HATs (Endsley, 2017) and is aligned with research on human teamwork demonstrating the benefits of team members, awareness of members’ diverse task‐related capabilities, and functional expertise for team effectiveness (Van Knippenberg & Schippers, 2007).

The need for situational awareness extends beyond agents' knowledge of human teammates' abilities, as research also underscores the need for *shared* situational awareness, that is, humans’ situational awareness of autonomous agents and vice versa, for effective human interaction and collective performance (Cummings & Guerlain, 2007; Endsley, 2017; Grimm, Demir, Gorman, & Cooke, 2018a, 2018b; Salmon et al., 2009). Humans’ situational awareness strongly influences the degree to which they need to oversee the autonomous agents (Boardman & Butcher, 2019), and autonomous agents also need to maintain a model of the state of their human teammates to perform their tasks (Barnes & Van Dyne, 2009; Carroll et al., 2019; Chakraborti, Kambhampati, Scheutz, & Zhang, 2017). In one study, autonomous monitoring of excessive workload or insufficient training prompted members to shift tasks to optimize team performance (Dierdorff, Fisher, & Rubin, 2019; Dorneich et al., 2017). In order to exert autonomy in a timely and appropriate manner, the agents need to formulate a mental model that refers to team members’ mental model and perception of current states, situational dynamics, and contextual cues. That is, agents can have preprogrammed mental models that script heuristic responses to an inventory of human behaviors, or if more advanced they can develop a machine theory of the human mind to adapt to the situation. Extant research on teamwork shows that members can anticipate and predict the needs of others when they have shared mental models, which is important for supporting mutual coordination (Goodwin, Blacksmith, & Coats, 2018) and underscores the need for this capability development in HATs.

As in regular human teams, feedback mechanisms play a significant role in HATs in enabling team members to monitor, evaluate their task performance, and provide opportunities to improve it (Salas, Dickinson, Converse, & Tannenbaum, 1992; Sottilare et al., 2018). Additionally, feedback processes help team members share experiences and develop mutual trust (Cuevas, Fiore, Caldwell, & Strater, 2007; Fan & Yen, 2011; Fan et al., 2008). Large bodies of research on HATs found that human–human teams outperform HATs due to more efficient information sharing among human teammates compared to HATs (Cooke et al., 2016; Demir, Cooke, & Amazeen, 2018; Demir, McNeese, & Cooke, 2018; Demir, Likens, Cooke, Amazeen, & McNeese, 2019; Demir, McNeese, & Cooke, 2016; N. J. McNeese et al., 2018; Myers et al., 2019) as well as better organization and adaptation (Grimm et al., 2018b). Efficient information sharing does not always mean high communication frequency, as the latter can result in higher cognitive load, misunderstandings, and inefficiency and thus undermine performance (MacMillan, Entin, & Serfaty, 2004). Hence, the investigation of communication frequency and quality related to team outcomes has become an essential branch of HAT research (Cooke et al., 2016; Demir, Cooke, & Amazeen, 2018; Demir, McNeese, & Cooke, 2016, Demir, Likens, Cooke, Amazeen, & McNeese, 2019; Demir, McNeese, & Cooke, 2018; N. J. McNeese et al., 2018).

Extant work on cognitive modeling has focused primarily on individual cognition and cognitive models of situation awareness to understand and emulate human behavior by representing the cognitive steps by which a task is performed (Adams, Tenney, & Pew, 1995; Bolstad et al., 2010; Endsley, 1995a, 1995b, 2015; Wickens, 2008, 2015). Very little work in this literature addresses team cognition, with only a handful of studies considering collective cognition in HATs (Cuevas et al., 2007; Saner, Bolstad, Gonzalez, & Cuevas, 2009; Wiltshire, Warta, Barber, & Fiore, 2017). Consequently, there are few models in this area for researchers to build on to model the cognition that AI teammates need to collaborate as part of a human team and make the kinds of contributions humans expect from a genuine team member.

Therefore, a critical need for supporting COHUMAIN research is work that can bridge between individual and collective‐level models of cognition and interaction. Such work is essential for enabling progress on developing sociocognitive architectures and guiding the design of AI agents.

### 3.2 Trust in human–AI collaboration

The interaction and communication that serves as the engine for developing collective cognition is also a basis for the formation and maintenance of human–AI trust, an essential quality which influences the ability of a sociotechnical system to achieve CI. Hence, advancing COHUMAIN research requires continuing to develop a deeper understanding of issues that facilitate or inhibit the formation, maintenance, and repair of trust between humans and AI collaborators. While human–AI trust is a new area of investigation, a long line of research on trust and trustworthiness in humans demonstrates the importance of demonstrating competence, benevolence, and integrity (McAllister, 1995).

In considering human–AI collaboration, an important question is examining the degree to which trust development is dependent on these same factors. If so, how are they demonstrated and established by the AI agent and by the human (Stanton & Jensen, 2021). In their review of the extant literature on human trust in AI, Glikson and Woolley (2020) concluded that the focus to date has been almost exclusively on dimensions related to competence, particularly reliability. Even the more recent work on explainable AI (T, Miller, 2019; Phillips, Hahn, Fontana, Broniatowski, & Przybocki, 2020) is largely focused on promoting human–AI trust by providing more comprehensive information on the reasons for various actions which will reinforce perceptions of its competence. Related work on AI and transparency (T. Miller, 2019) contributes to competence perceptions in a similar manner but also begins to connect to perceptions of integrity as well, where an agent is providing more demonstrable evidence of its reasoning and thereby allowing human users to observe that it is pursuing the goals that are intended, and nothing else. Issues of data privacy and disclosure continue to put pressure on perceptions of AI integrity as different groups of AI developers, users, and increasingly government and nonprofit agencies, and institutions get involved in establishing guidelines for disclosure and user control over private information.

One area of human–AI trust which has received considerably less attention relates to humans’ perceptions of agent benevolence and related affective bases of trust (Glikson & Woolley, 2020; McAllister, 1995). Much of the work to date in this area has focused on AI agent characteristics and user‐interface design, such as the level of embodiment of the agent and other attributes related to its identity, or how the agent looks and sounds, and the resulting impressions of users. However, the general patterns across existing studies demonstrate that these elements only influence initial impressions; even users who begin with a strong positive impression of agents typically exhibit a loss of trust, particularly when an agent's capabilities were not clearly presented, and the subsequent performance falls below users’ expectations (Glikson & Woolley, 2020). Beyond the issues of how AI agent features affect users' reactions, at least initially, very little is known about how human perceptions of AI benevolence develop or the degree to which human users perceive that their motives are aligned, and the agent is acting in their best interests and, possibly, even cares about them (Hancock et al., 2011). This is an important area for further development, as research on trust between humans, or even between humans and institutions, demonstrates that it is strongly influenced by perceivers’ assessment that their goals and motivations are aligned with those of the other party (be it a human, organization, or a non‐human actor) and the other party cares about them and is working toward the same outcomes.

Issues of trust repair in human–AI interaction are not yet well understood, but as AI agents become more involved in human collaboration, it will be important to understand how AI agents not only convey trustworthiness but also establish trust and detect whether trust has been broken. The value of adaptively calibrating and managing human–AI's dyadic trust dynamics has been explored to some degree in autonomous car driving and drone flying (Akash et al., 2020; Okamura & Yamada, 2020). This will be essential to developing a nuanced understanding of human–AI interaction as an ongoing relationship, one that develops and changes over time as the needs and capabilities of both parties change, and to identify ways of communicating and resolving conflict that enable the relationship to evolve as well. For example, an AI agent can establish trustworthiness by being transparent about whether the capabilities needed to help pursue specific goals are or are not within their established repertoire. Such a level of proactive communication in any relationship reduces fears of deception and consistently serves to prevent conflict and promote trust. The process of developing detailed models of how AI agents establish and maintain trust may actually change our basic understanding of trust development and repair in all relationships. This capability will also draw extensively on “machine theory of human mind” (MToHM) as an extension of the human ToM and thus move related research forward as well.

### 3.3 Machine theory of mind

Recent advances in AI and computation have led to increased development of learning ToM agents, which are AI agents that can predict cognitive states (e.g., desires, intentions, beliefs) of other agents. This work follows existing research that has been focusing almost exclusively on investigating how a machine can predict another machine's cognition (MToMM—machine theory of machine mind) and how a human is able to understand a machine's cognition (HToMM—human theory of machine mind). Much of this work resides in the cognitive science literature. Here, humans or ToM‐enabled AI agents serve in the role of an “observer” who observes the actions of an AI “actor” and make predictions about the actor's mental state.

Traditionally, ToM agents have been developed using the plan and goal recognition algorithms (Geib & Goldman, 2009; Kautz & Allen, 1986); however, such an approach requires a detailed description of the domain to use as a basis for modeling the full range of goals and plans. The Bayesian ToM observer (Baker, Jara‐Ettinger, Saxe, & Tenenbaum, 2017; Baker, Saxe, & Tenenbaum, 2011), one of the prominent computational ToM frameworks, is developed by constructing a model of the actor's cognition by relying on the assumption that the actor will take actions that maximize its utility based on partial observations. This assumption, however, tends to deviate from actual human behavior in many decision‐making tasks showing that humans are often boundedly rational (Kahneman, Slovic, & Tversky, 1982; Simon, 1997) and that their decisions are constrained by human cognitive capabilities for storing and retrieving information from memory (Gonzalez et al., 2003). Recently, the deep learning approach to ToM has received extensive attention since it leverages the computational efficiency and the architecture of neural networks (Oguntola, Hughes, & Sycara, 2021; Rabinowitz et al., 2018). Despite the robustness of the neural network ToM models, their agreement with human observers’ judgments remains unclear.

Despite many promising developments, these aforementioned ToM models do not capture human biases such as bounded rationality and fall short of the same significant attention or limited processing capacity of humans. This suggests the need for a cognitive approach to ToM. For instance, in an attempt to simulate human ToM representation, Nguyen and Gonzalez (2022) developed a cognitive model of the observer (CogToM), which relies on the cognitive theory of decisions from experience, IBLT. As such, the CogToM observer model is boundedly rational by considering decisions constrained within the limitations of human memory (e.g., recency and frequency biases of information and errors in the retrieval of information). Experimental results demonstrate that the CogToM observer can make inferences that are in agreement with human observers’ judgments on the same task. At this stage, CogToM has not yet been applied to predict humans’ behavior as a player in the observed environment.

Given the above, research on extending the understanding from MToM to developing a deeper knowledge of how a machine can model and predict the cognitive states of genuine humans (MToHM) is an important and relatively underexplored area. Furthermore, there is a lack of computational ToM methods that have direct applicability to COHUMAIN research, wherein the learning AI agents must not only learn to form mental states such as beliefs about the knowledge possessed by their collaborators (i.e., humans or autonomous AI agents) but also leverage such beliefs to make appropriate decisions on which actions to take.

Hence, MToHM and HToMM are foundational to the development of well‐functioning sociocognitive architecture. Importantly, AI agents with cognitive architectures that support the development of both MToHM and HToMM will be especially important for supporting the emergence of CI and thus central to COHUMAIN research.

## 4 A sociocognitive architecture for COHUMAIN: TSM‐CI

In the previous sections, we outlined four core problems for COHUMAIN research and advocated for the use of sociocognitive architectures as a vehicle for a holistic approach to its design and development. We then reviewed the relevant literature on human–machine interaction, human–AI trust, and machine ToM which supply key inputs to solving the core problems of this nascent domain. In this section, we describe a possible sociocognitive architecture, the TSM‐CI (Gupta & Woolley, 2021), and extend it to the COHUMAIN domain by discussing how AI agents can augment its core processes. Following this, in the final section, we discuss the value of choosing a compatible cognitive architecture and illustrate it in this context by describing work on instance‐based learning theory.

For decades, research on intelligence has studied the functions that enable systems to adapt and accomplish goals in a wide range of environments that vary in complexity (Legg & Hutter, 2007). Some parallels across studies of intelligence in different domains suggest that intelligence in any system— biological, technological, or hybrid—requires the fulfillment of certain memory, attention, and reasoning functions. In parallel, over the last few decades, there is increasing recognition in the management literature that human organizations operate less as static structures, as traditionally portrayed, and more as complex adaptive systems, requiring a deeper understanding of the process dynamics that underlie different modes of organizing (Arrow, McGrath, & Berdahl, 2000). These parallel developments in the intelligence and management literature have been reflected in the increasingly common inclusion of concepts originating in intelligence in organizational theory (Csaszar & Steinberger, 2021).

TSM‐CI explicitly integrates research on intelligence across fields with extant work on teamwork and collaboration to support the premise that CI is fostered by the emergence and ongoing adaptation of three interlocking sociocognitive systems centered around collective memory, attention, and reasoning (Gupta & Woolley, 2021). TSM‐CI is a process model describing how individual agent‐level cognitive functions, between‐member metacognitive processes, and between‐member transactive processes interact and lead to the formation of three dynamically stable sociocognitive systems. When strong transactive memory, attention, and reasoning systems develop, they enable collaborators to overcome the limits of individual cognitive capacity and expand the collective's total memory, attention, and reasoning capacity. The concomitant alignment of goals and mental states creates a readiness for coordinated action as an adaptive response to environmental changes.

**Fig. 2:**
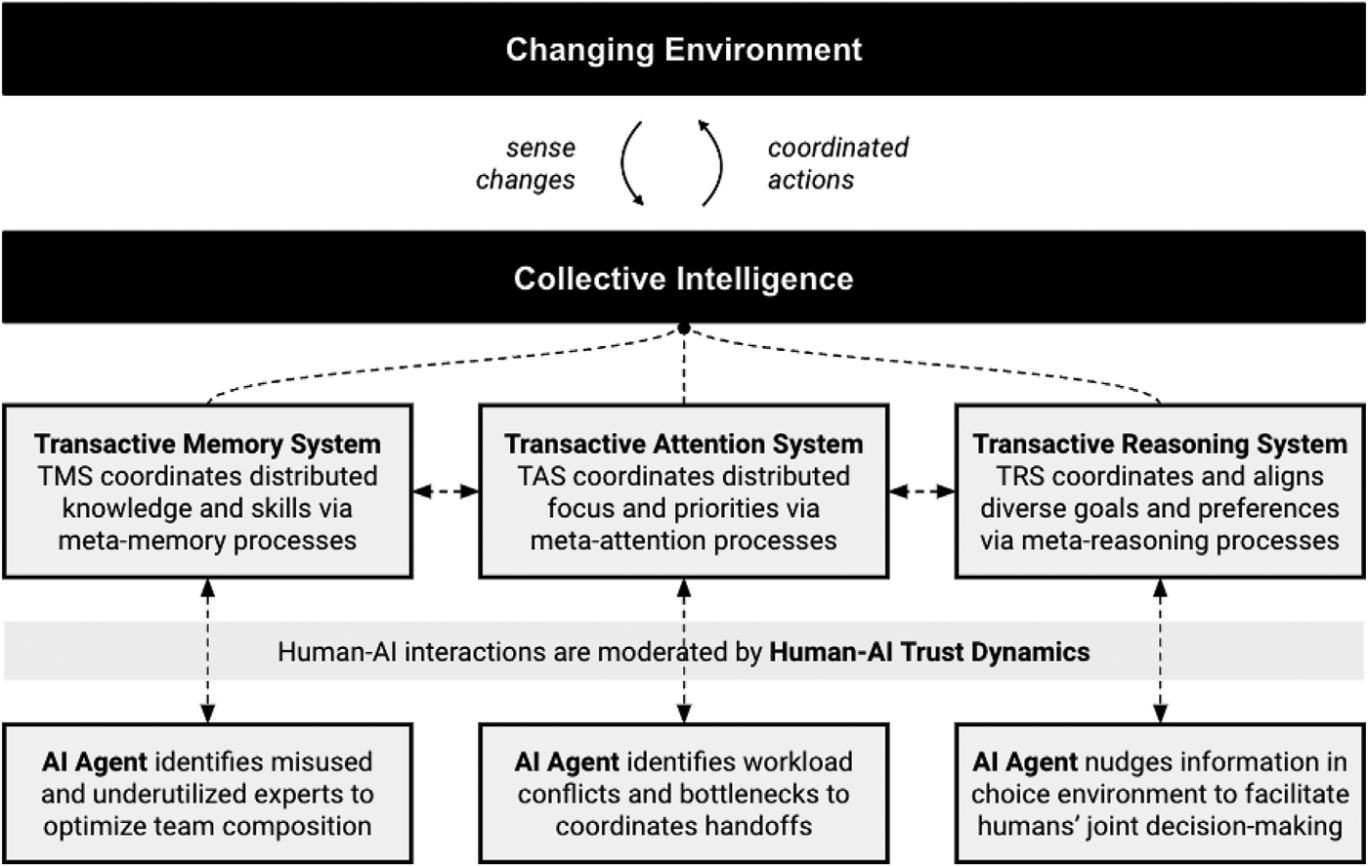
Overview of the extended transactive systems model. The collective memory, attention, and reasoning systems emerge due to between‐member processes, and together, they adaptively respond to a changing environment.

Here, we provide a brief overview of the transactive memory, attention, and reasoning systems that form the foundation of the TSM‐CI. As we describe each component, we also discuss opportunities for AI‐based agents to facilitate the development and maintenance of associated functions and enhance CI (see Fig. 2).

### 4.1 Transactive memory system

A transactive memory system (TMS) is one of the three sociocognitive functions in the TSM‐CI that addresses two of the core problems of COHUMAIN. Specifically, TMS is a dynamic system of processes through which collaborator's knowledge and skills (i.e., cognitive resources) and their beliefs about who knows what (i.e., metamemory) are dynamically updated to facilitate the allocation and retrieval of knowledge to and from the most appropriate collaborator (Wegner, 1987, 1995). This enables an effective response in the face of changing knowledge interdependencies in the environment. The concept of TMS was initially developed in the context of couples in close relationships by Wegner (1987), but then extended to the group level and associated with team performance (Ren & Argote, 2011) as well as CI (Y. J. Kim, Aggarwal, & Woolley, 2016).

The foundational component of TMS is the collaborators’ individual memory systems. Its function is to (1) reliably store knowledge, thereby building member skills and (2) accurately retrieve and apply this stored knowledge to complete tasks successfully. Technology plays a significant role in augmenting individual human memory. Search engines and online knowledge repositories are resources that individuals regularly use as external memory aids—both for their information searches, as well as the proactive alerts that make them aware of new knowledge. This access has already changed individual human behavior.

Research demonstrates that when individuals expect to have future access to information they found online, they have lower recall rates for the information itself and enhanced recall instead of where on the internet to access it (Sparrow, Liu, & Wegner, 2011). The encoding of the location of the information, also referred to as metaknowledge, instead of the contents of the information itself, is formed in metamemory. Metaknowledge encoding also occurs with respect to the knowledge and skills of other collaborators. As TMS develops, with multiple experiences successfully updating, allocating, and retrieving information from one another, collaborators’ knowledge becomes more differentiated and specializations emerge. Moreover, repeated interactions also establish trust and credibility with others. Both of these patterns are validated markers of a well‐developed TMS (Ren & Argote, 2011).

### 4.2 The role of AI collaborators in TMS

The processes described for developing a strong collective memory via TMS is one that has been studied mostly in all‐human groups but could involve a mix of human and AI collaborators, In addition to the role of AI in augmenting individual memory mentioned above, AI‐based teammates could also enhance collective memory by speeding up the process of learning who knows what and facilitating the allocation of tasks and information. AI could also connect individuals needing to learn a skill to someone who can teach them or facilitate adaptation in response to membership changes by helping realign specializations with a new configuration of skillsets.

While the possibilities resulting from a deeper integration of AI into human teams are exciting, a small cautionary note is also important to consider, related back to the earlier discussion of human–AI trust. On the one hand, a certain level of trust will be required in order for humans to allow AI to have access to the level of knowledge necessary to facilitate collective memory in some of the ways described. On the other hand, it is possible that humans might come to trust such AI teammates too much and become too reliant on their facilitation in ways that undercut individual memory formation as well as the formation of collective memory. For example, Gupta and Woolley (2018) found that a digital dashboard that tracked information about team members’ knowledge and specialization actually undermined collective cognition and performance when the number of team members was small enough that collaborators could have kept track without such a tool. This underscores the importance of a holistic sociocognitive architecture that integrates AI‐based tools into teams in a manner that enhances individual and collective cognition rather than detracting from it.

### 4.3 Transactive attention system

In any collective, the total attention of collaborators creates the upper bound of the system's capacity to handle information (Simon, 1973). In addition to coordinating collective memory consisting of distributed knowledge and skills, collectives must also distribute their members’ limited and often fragmented attention to accomplish interdependent work per its importance to the group. Just as was described in the case of a TMS, a transactive attention system (TAS) is a process for coordinating collaborators’ attentional resources and aligning their shared beliefs about each other (meta‐attention) to produce collective cognition—in this case, jointly allocating and retrieving their collective attention. In the TSM‐CI sociocognitive architecture, TAS is intended to complement TMS, in that the two mutually regulate and efficiently balance the use of both members’ knowledge and attention.

The foundational component of TAS is the individual attention system, whose job it is to reliably filter, chunk, and process information to complete tasks successfully (Knudsen, 2007). It is often the case that individuals have different tasks competing for attention, requiring them to switch among them frequently. A person's meta‐attention provides globalized cognitive control to help them expertly navigate situations involving choice among multiple tasks (Lavie, Hirst, de Fockert, & Viding, 2004; P. H. Miller & Bigi, 1979) for minimizing switching costs. A well‐developed meta‐attention also allows collaborators to develop a joint awareness of each other's workload and availabilities to help reduce the coordination costs associated with interdependent tasks. For instance, groups with a strong TAS will exhibit organized patterns of synchronous attention, sometimes manifesting as “burstiness,” where periods of independent work are punctuated by periods where members are highly responsive to each others’ requests (Mayo & Woolley, 2021; Riedl & Woolley, 2017). These bursty patterns are interpreted as evidence that collaborators have developed routines that support the need for synchronous coordination while also enabling collaborators to flexibly juggle other responsibilities without the expectation of constant availability.

#### 4.3.1 The role of AI collaborators in TAS

Just as there are AI‐based tools that enhance individual memory, many tools are commonly used to augment individual attention. Calendar‐based algorithms and personal digital assistants prompt individuals with reminders depending on time or location based on the triangulation of information or instructions. Tools that filter incoming communications based on past behavior to limit interruptions or distractions are also becoming more widely used. However, very few tools exist to help develop and maintain meta‐attention or foster collective attention by facilitating TAS. Building tools to do so would be a great way to further develop COHUMAIN research. A key hurdle for developing TAS among human collaborators is a broad understanding of collaborators’ workloads, plans, and progress vis‐a‐vis individual and collective priorities. Tracking all of those details would result in information overload, but without a way to do so it is difficult for collaborators to know enough to manage collective attention. An AI‐based teammate could help make information available and processes more visible at the level of detail necessary to enable ongoing coordination. Certainly, AI tools could take over and “manage” the entire task flow of all collaborators; however, tools that deprive humans of autonomy and remove the need for mutual attention by handling work assignments can undermine motivation and integration of teamwork products, ultimately impeding performance (Woolley, Gerbasi, Chabris, Kosslyn, & Hackman, 2008). Therefore, just as highly sophisticated AI tools can inadvertently undermine collective memory, micromanaging the coordination of work can similarly undermine collective attention.

Some sophisticated approaches to managing collective attention are being developed in the context of crowd‐based work. For example, studies using the experimental platform “Foundry” examine ways to automate the structure of temporary online (or “flash”) teams to collaborate on more creative, open‐ended projects than are generally conducted using crowd workers online (Retelny et al., 2014; Valentine et al., 2017). This temporary teaming model facilitates the workflow of short‐term decomposable projects in relatively small groups of collaborators. Solving a poorly specified problem over longer time horizons will likely need different strategies. Thus it is essential in developing sociocognitive architectures for COHUMAIN that researchers remain cognizant of supporting the development of human collective cognition and avoids introducing AI tools that displace it.

### 4.4 Transactive reasoning system

Thus far, we have described two of the systems forming TSM‐CI sociocognitive architecture, TMS and TAS, which both function to enable the efficient use of collaborators’ distributed memory and attentional resources to achieve coordinated action. However, while TMS and TAS serve to enhance the efficient use of cognitive resources, they do not address another essential element which is the alignment of cognitive states (goals, preferences, and beliefs) with outcomes valued by collaborators and/or the environment. Tracking changes in the environment with implications for the relative value of different goals is critical to a system's ability to maintain itself in addition to being productive. Collective reasoning serves these functions: it evaluates collective goals in the context of a constantly changing environment to ensure the pursuit of those with the greatest value with respect to related resource uncertainties and the alignment between individual and collective goals (Bacharach, 1999; Locke & Latham, 1990).

An individual's reasoning system maintains a hierarchy of cognitive structures that represent a set of goals along with a set of tasks that are their means of attainment. Goals are typically associated with motivational needs, and continuously fulfilling them sustains progress toward valued distal goals (Bandura, 1991). While goal hierarchies maximize short‐term rewards and generate goal commitment, individual metareasoning is needed to achieve metacognitive control that maximizes longer‐term rewards. Metareasoning involves monitoring and determining whether to continue, switch strategies, or terminate pursuing the current set of proximal goals to avoid local reward maximas (Ackerman & Thompson, 2017). Individuals with well‐developed metareasoning are able to effectively adapt to the changing situation by reconfiguring their goal hierarchies and ensuring reward maximization. Research on COHUMAIN can build models that represent how individuals break down tasks and build goal hierarchies which facilitate metareasoning by enabling the comparison across goals.

In human groups, the transactive reasoning system (TRS) emerges as a consequence of humans’ ability to infer, understand, and reason about others’ goals and motivations. This metareasoning ability is necessary for the collective exploration of diverse goals and the selection of joint priorities via a process of negotiation among collaborators. When done well, the resulting alignment of collective actions with individual goals as well as the adoption of transactive, that is, other‐ and system‐oriented goals, elicits commitment toward the collective as a whole (Fitzsimons & Finkel, 2018). Consequently, two markers of a highly effective TRS are a high level of collective effort and strong individual commitment. These, in turn, facilitate the ongoing updating of goals, as highly motivated individuals are better at recognizing and creating opportunities from their environment (Carsrud, Brännback, Elfving, & Brandt, 2009). The guidance on goals and priorities from the TRS, in turn, shapes decisions about resources enabling the TAS and TMS processes to execute collective actions effectively.

#### 4.4.1 The role of AI collaborators in TRS

Unlike the current context for TMS and TAS, there are very few tools at the individual or collective level to facilitate collective reasoning. There are a handful of technologies, including AI‐enabled tools, which nudge the information in the collective choice environments to facilitate the human members' joint decision‐making. Decision support systems can help to structure and facilitate the surfacing and exchange of different points of view and identification of member preferences to guide decision‐making (Chidambaram, Summers, Miranda, Young, & Bostrom, 2020). Algorithm‐assisted prediction and auction markets can facilitate negotiation around limited resources and maximization of joint outcomes via market‐like bidding systems (Malone et al., 2017). Recent developments incorporate models of “swarm intelligence” to gauge collective sentiment and provide a high‐level view to all members to help facilitate consensus (Rosenberg, Willcox, Palosuo, & Mani, 2021). However, there are additional opportunities for AI to initiate such exchanges and prompt collective reasoning based on indicators that members may have misaligned goals, such as when the level of member engagement declines based on the pace of activity and emotional states (Van Kleef, Homan, & Cheshin, 2012).

In this section, we described the three sociocognitive systems that together form the sociocognitive architecture guided by the TSM‐CI. In extending its possibilities for shaping COHUMAIN, we discussed some examples of ways AI‐based teammates could enhance the three essential functions. In the next and final section, we make a case for designing AI agents with cognitive architectures that are compatible with the sociocognitive architecture of the collective. In doing so, we discuss a learning‐based cognitive architecture, specifically IBLT, which we identified to be most compatible with extended TSM‐CI.

## 5 Illustrating a compatible learning‐based cognitive architecture for agent cognition

A core premise of COHUMAIN is that CI is manifest when environmental changes trigger a coordinated response, where the response is a collection of member interactions (human–human, human–AI, and AI–AI) working in tandem. Achieving this requires the coordination of distributed cognitive resources and the alignment of diverse mental states to develop collective cognition that results in intelligent behavior. In the TSM‐CI sociocognitive architecture, this coordinated response results from the dynamic regulation of collective memory, attention, and reasoning systems.

Ideally, one would want all the independent AI agents facilitating and improving various aspects of the collective's TMS, TAS, and TRS to operate as one holistic system. In an integrated AI system, all the AI agents could seamlessly feed into each other to ensure the maintenance of CI. Yet, as technology develops and AI agents get good at solving specific coordination problems, one should reasonably expect collectives to comprise multiple AI agents that do not necessarily play well with each other. Hence, it matters the kind of design choices companies and developers make when building the cognitive architecture of their AI agents.

Some cognitive architectures are better suited for interfacing with a given sociocognitive architecture in that the paradigm guiding an AI agent's internal representations aligns with the intermember processes driving the larger sociocognitive architecture of the collective they are participating in. Furthermore, the AI agent needs to be capable of generating human‐understandable explanations of their ongoing actions and underlying internal cognitive states as well as infer the humans’ cognitive states from their communication to enable human–AI collaboration. Thus, a cognitive architecture that has the most congruent mechanisms for representing ToM is likely to be more successful.

*We claim that investigating the features across various cognitive architectures used to develop AI agents and what makes them most compatible with a given sociocognitive architecture is an important agenda item for realizing the integration envisioned for COHUMAIN research*. Here, we illustrate this by discussing work on a learning‐based cognitive architecture, specifically IBLT, which results in models that are highly compatible with the extended TSM‐CI sociocognitive architecture.

### 5.1 Learning‐based models of individual cognition

Information‐processing theories are perhaps best suited to describe individual cognition as an interaction between an information‐processing system (e.g., the human) and a task environment (Simon, 1978). The information‐processing system involves a set of complex processes, including perception and sensorial information, attention, a memory system (e.g., working memory and long‐term memory), decision‐making and problem‐solving, motor action components, and feedback or learning processes. Each of these elements is a complex system in itself. Furthermore, the information‐processing system is adaptive and capable of adjusting to dynamic changes in the environment and improving behavior through learning (Gonzalez, 2013).

One aspect of individual cognition that is essential for developing a sociocognitive architecture of COHUMAIN is the individual learning system, which converts experience in the environment into the individual's current level of understanding of the task and the consequences of their actions. Learning is an essential process in the context of collaboration, as the knowledge individuals gain from experience with others in the environment influences how the individual formulates their goal and where they direct their attention in the task environment. Learning is achieved by processing experiences stored in the individual's memory system, which is commonly defined by its capacity, duration, and speed (e.g., short‐term and long‐term memory), and other factors. However, beyond these factors, memory is a complex dynamic system that functions largely based on a number of intricate processes of storage, retrieval, organization, recognition, and updates (Simon, 1976). Decades of work by psychologists have resulted in an enormous amount of research on learning and human memory as an information‐processing system (e.g., Anderson & Milson, 1989; Anderson & Schooler, 1991; Anderson, Reder, & Lebiere, 1996). There is also a large and growing body of work on the computational representation of such memory processes (e.g., Borst & Anderson, 2013; Hintzman, 1984; Lovett, Reder & Lebiere, 1999; O'Reilly, Braver, & Cohen, 1999). Given the infeasibility of a comprehensive review of this large literature, here we will focus on the most relevant elements of individual cognitive models that use learning as the basis for taking action and making decisions. These are also chosen for their congruence with the larger transactive systems model, which relies on memory and attention processes at the collective level.

Memory‐based models that represent and explain the dynamics of human decisions and learning from experience are highly specialized types of models that rely on theories of choice and cognitive theories of memory processes (see Hertwig, 2015 for a discussion of models of decisions from experience). The general idea from theories of choice is that decisions are made according to some form of the expected value of the alternatives considered based on a combination of the value of each option and its probability of occurring. This is an idea that dates back to Bernoulli (1700–1782), and that was formalized in theories of expected utility and psychological theories such as prospect theory (Kahneman & Tversky, 1979). These ideas have also informed models of experience‐based choice, for which the values of the alternatives are formed from the decision makers’ experience (Gonzalez & Dutt, 2011; Gonzalez et al., 2003; Nguyen et al., 2023).

The process models of decisions from experience are associative learning models that conceptualize choice as a dynamic learning process that relies on the associations of behavior and outcome contingent on a particular situation. Many common models fall into this modeling approach, including reinforcement learning (Sutton & Barto, 1998), mathematical models of choice (Denrell, 2007; March, 1996), and cognitive models of decisions from experience (Erev et al., 2010; Gonzalez & Dutt, 2011; Lejarraga, Dutt, & Gonzalez, 2012). Here we focus on IBLT, a theory of decisions from experience that articulates the general cognitive processes of experiential choice (Hertwig, 2015), which results in models that generalize and outperform the models based on many other theories of decisions from experience proposed in modeling competitions (Erev et al., 2010).

### 5.2 Instance‐based learning theory

IBLT emerged from the need to explain the process of dynamic decision‐making, where a sequence of interdependent decisions are made sequentially and over time (Gonzalez et al., 2003). IBLT provides a single general algorithm and mathematical formulation of memory retrieval that relies on the well‐known ACT‐R cognitive architecture (Anderson & Lebiere, 2014). The theory proposes a representation of decisions in the form of instances, which are triplets involving the state, actions, and utilities. States are a representation of the features of the situation in a task, actions are decisions an agent makes in such states, and utilities are the expectations the agent generates or the outcomes the agent receives from performing such actions. IBLT also provides a process for generating accumulated value (expectation from experience) for each choice alternative based on a mechanism called blending, which is a function of the payoffs experienced and the probability of the agent retrieving those instances from memory (Gonzalez & Dutt, 2011; Lejarraga et al., 2012).

IBLT is particularly useful in the context of the transactive systems model as it encodes actions that the AI agent takes (in the world or interactions with others) as experiences in its memory. And, owing to its learning mechanism, specific sets of action, or decision sequences emerge (akin to attentional hierarchy) that prove to be most productive given its reward function. Importantly, we can also query IBLT models for experiences that constitute these productive decision sequences.

Recently, the instance–based learning (IBL) algorithm has been applied to multistate grid world tasks (Nguyen & Gonzalez, 2020, 2022) and to tasks in which multidimensional state‐action‐utility representations are required to build real‐time interactivity between models and humans (Nguyen, Phan, & Gonzalez, 2022). With the increased use of IBLT in generating models on tasks of greater complexity and in multiple domains, models in tasks that involve multiple players are also becoming more common. Initial theoretical developments of IBLT in this direction involved two‐person game theoretical models (Gonzalez, Ben‐Asher, Martin, & Dutt, 2015). More recently, other interesting representations have been proposed, including the ability to represent a ToM (Nguyen & Gonzalez, 2022).

ToM refers to the ability to infer and interpret the beliefs, desires, and intentions of others (Premack & Woodruff, 1978; Rusch, Steixner‐Kumar, Doshi, Spezio, & Gläscher, 2020), and it is an essential component of human learning and social cognition, including the acquisition of social norms and social beliefs (MacLean, 2016). Nguyen and Gonzalez (2022) address the challenge of creating computational models of machine theory of mind (MToM) and verify whether those models are able to emulate human ToM. Creating computational representations of MToM provides an important foundation for AI research, and researchers have primarily used one of two prominent computational approaches based on Bayesian models (Baker et al., 2011, 2017) or deep learning (Rabinowitz et al., 2018). Nguyen and Gonzalez (2022) additionally demonstrate how an IBL model can provide a more accurate representation of human ToM compared to the other approaches, which is confirmed by experimental evidence in which humans make predictions about others’ actions that are consistent with the IBL model's predictions. IBL models can also predict the false beliefs of an acting agent, another ability that is essential to ToM (Baron‐Cohen, Leslie, & Frith, 1985).

In summary, extant work on learning‐based cognitive modeling, specifically IBLT, provides an important understanding of how individual agents generate knowledge by learning from the environment that helps them attend to their goals and direct their actions. In addition, researchers can use the same mechanisms for building human‐like ToM models by observing others' behavior. Thus, IBLT supplies critical building blocks for modeling shared cognition processes—memory, attention, and reasoning— central to TSM‐CI.

More generally, a well‐designed sociocognitive architecture that successfully leads to the emergence of collective human–machine intelligence is likely to comprise AI agents with internal mechanisms for dealing with interactions and MToM that easily interfaces with the sociocognitive mechanisms. Thus, we assert that evaluating various combinations of cognitive and sociocognitive architectures for compatibility will be critical for advancing COHUMAIN research.

## 6 Conclusion

In this paper, in addition to proposing a guiding research agenda for studying sociocognitive architectures that allow collective human–machine intelligence to emerge, we also present one. Specifically, we describe and extend the TSM‐CI—an integrated and interdisciplinary systems approach—for building a sociocognitive architecture that breaks down the phenomenon of CI into three coregulatory systems for coordinating collective memory, attention, and reasoning. We also highlight the value of using AI agents with cognitive architectures that align with the sociocognitive one. Specifically, discussing IBLT, a particularly compatible cognitive architecture, for developing the AI agents within the transactive systems model.

The early‐stage development in the field of AI (1970s through the 1990s) significantly benefited from the numerous and diverse attempts at proposing and testing a variety of cognitive architectures. Hence, we think there is tremendous value in creating many different approaches to theorize, design, and test sociocognitive architectures that deal with the dynamics of how humans and machines work together. This demands playing around with models that integrate and expand the research findings from management and behavioral sciences and design lessons from cognitive sciences and AI. We take this opportunity to call upon researchers across fields to not only engage with our proposal but also develop new and varied sociocognitive architectures to help unlock the potential impact of COHUMAIN.

## Note

1 In this paper, we refer to autonomous machines or AI agents to be *autonomous* only in the sense of independently perceiving and processing information from a changing environment as well as teammates to generate plausible actions that are aligned with their designed goals. While the agents may generate internal goal hierarchies at the task level, unlike humans, these AI agents do not have complete goal autonomy. That is, they lack intrinsic motivation and the overarching or ultimate goal (or a set of AI system constraints) that they optimize for are chosen by their developers. For example, a search engine generates results that are “relevant” to the user and “profitable” for the company. In this way, AI agents are developed with desired goal that may or may not align with their human collaborators. And may vary in how flexibly they will adjust those goal states in response to their interaction. As these goals/values are ostensibly hidden it is beneficial for the humans in the collective to understand and use them with caution. Thanks to an anonymous reviewer for pointing out this distinction between autonomous action and goal autonomy.
